# Emerging Role of Exosomal Long Non-coding RNAs in Spaceflight-Associated Risks in Astronauts

**DOI:** 10.3389/fgene.2021.812188

**Published:** 2022-01-17

**Authors:** Malik Bisserier, Nathaniel Saffran, Agnieszka Brojakowska, Aimy Sebastian, Angela Clare Evans, Matthew A. Coleman, Kenneth Walsh, Paul J. Mills, Venkata Naga Srikanth Garikipati, Arsen Arakelyan, Lahouaria Hadri, David A. Goukassian

**Affiliations:** ^1^ Cardiovascular Research Institute, Icahn School of Medicine at Mount Sinai, New York, NY, United States; ^2^ Physical and Life Sciences Directorate, Lawrence Livermore National Laboratory, Livermore, CA, United States; ^3^ Department of Radiation Oncology, University of California, Davis, Sacramento, CA, United States; ^4^ School of Medicine, University of Virginia, Charlottesville, VA, United States; ^5^ Center of Excellence for Research and Training in Integrative Health, University of California, San Diego, San Diego, CA, United States; ^6^ Department of Emergency Medicine, Dorothy M. Davis Heart Lung and Research Institute, Ohio State University Wexner Medical Center, Columbus, OH, United States; ^7^ Bioinformatics Group, The Institute of Molecular Biology, The National Academy of Sciences of the Republic of Armenia, Yerevan, Armenia

**Keywords:** exosomes, lncRNA, biomarkers, astronauts, spaceflight

## Abstract

During spaceflight, astronauts are exposed to multiple unique environmental factors, particularly microgravity and ionizing radiation, that can cause a range of harmful health consequences. Over the past decades, increasing evidence demonstrates that the space environment can induce changes in gene expression and RNA processing. Long non-coding RNA (lncRNA) represent an emerging area of focus in molecular biology as they modulate chromatin structure and function, the transcription of neighboring genes, and affect RNA splicing, stability, and translation. They have been implicated in cancer development and associated with diverse cardiovascular conditions and associated risk factors. However, their role on astronauts’ health after spaceflight remains poorly understood. In this perspective article, we provide new insights into the potential role of exosomal lncRNA after spaceflight. We analyzed the transcriptional profile of exosomes isolated from peripheral blood plasma of three astronauts who flew on various Shuttle missions between 1998–2001 by RNA-sequencing. Computational analysis of the transcriptome of these exosomes identified 27 differentially expressed lncRNAs with a Log_2_ fold change, with molecular, cellular, and clinical implications.

## Introduction

Astronauts are exposed to various unique environmental factors during spaceflight, including isolation, confinement, acceleration at launch, sleep deprivation, microgravity, and space radiation, which have been associated with a variety of biological changes and a range of harmful health consequences. These adverse effects include: neurological disorders (neurovestibular impairment, vision alteration, cognitive, and behavioral alterations) (Payne et al., 2007; Lawley et al., 2017; Garrett-Bakelman et al., 2019), gastrointestinal complications (alteration of the gut microbiome, reduced gastrointestinal motility) (Amidon et al., 1991; Siddiqui et al., 2021), immune disorders (impaired immune response, reactivation of the latent viruses, rheumatoid arthritis) (Sonnenfeld, 2002; Mehta et al., 2018), adverse renal manifestations (formation of kidney stones, acute renal retention) (Okada et al., 2011), cardiovascular events (Bisserier et al., 2021), and body composition disorders (bone demineralization, fluid redistribution muscle atrophy) (Zobel et al., 2012; Tanaka et al., 2017; Vernice et al., 2020). Retrospective studies suggest that, compared to non-flight and low Earth orbit (LEO) astronauts, the lifetime risks associated with cardiovascular morbidity/mortality are 4-5 fold higher in Apollo lunar astronauts that traveled into deep space (Delp et al., 2016). As of today, only 12 astronauts have traveled beyond the Earth’s protective geomagnetic Van Allen radiation belt. Over the past decades, increasing studies have highlighted the importance of further investigating the effects of space travel-associated stressors on astronauts’ health.

A large body of evidence from studies conducted on the International Space Station (ISS) in prokaryotic or eukaryotic cells and fruit flies suggests that the space environment may negatively impact astronauts’ health. On Earth and in LEO onboard the ISS, the human body is protected from high-energy particles (protons, electrons, and most nuclei of the periodic table of elements) by Earth’s geomagnetic sphere. However, outside of LEO, astronauts can be exposed to high levels of Solar Particle Events (SPEs) and Galactic Cosmic Rays (GCRs) (Chancellor et al., 2014), which represent two primary sources of ionizing radiation in space (Chancellor et al., 2014). SPEs consist of highly energized particles (primarily protons) emitted by the Sun during a solar flare. In contrast, GCRs have multiple sources of origin (the Sun, outside the Solar System, and distant galaxies) and are composed of 87% hydrogen ions, 12% helium ions, and 1–2% are particles with high atomic numbers and high energy nuclei (HZE) (Mewaldt, 1994; Moreno-Villanueva et al., 2017). Both SPEs and GCRs represent a significant radiation hazard to astronauts. Previous studies showed that ionizing radiation in space might be associated with adverse effects such as flashing lights from retinal exposure (Budinger et al., 1972), early-onset cataracts (Cucinotta et al., 2001), and maladaptive behavior from central nervous system damage (Cekanaviciute et al., 2018). In addition to space radiation, astronauts are also exposed to microgravity, which is also associated with health effects such as bone mineral loss (Grimm et al., 2016), changes in cerebral hemodynamics (Du et al., 2021), redistribution of blood toward the head (Shen and Frishman, 2019), and reduction in muscle mass (Tanaka et al., 2017).

Previous studies suggested that spaceflight may modulate gene expression in the whole blood of astronauts (Barrila et al., 2016). Our group and others have previously demonstrated that exposure to ionizing radiation or microgravity significantly affects the transcriptomic profile in human cells and mouse heart tissue (Carmeliet and Bouillon, 1999; Taylor et al., 2002; Coleman et al., 2015; Barrila et al., 2016; Garikipati et al., 2021). Similarly, previous microarray analysis revealed variations in the gene-expression patterns across individual crew members, partly due to the temporal variability in gene-expression patterns in human blood (Whitney et al., 2003; Barrila et al., 2016). Further studies in mice and human cells (peripheral blood mononuclear cells, lymphoblastoid TK6 cells) have shown increases in reactive oxygen species (ROS)-induced DNA damage and significant changes in the transcriptome profile due to microgravity and radiation (Yatagai et al., 2019; Fu et al., 2020; Bisserier et al., 2021). More recently, the National Aeronautics and Space Administration’s Twin Study revealed a constellation of effects from microgravity and space radiation on the human genome. Comparison of the genomes of monozygotic twins revealed 481 genes were differentially expressed post-spaceflight compared to pre-flight (Garrett-Bakelman et al., 2019). The authors also found increased circulating cell‐free mtDNA (cf‐mtDNA) levels, which was associated with higher mitochondrial gene expression and an increase in two urinary markers of oxidative stress during the flight. Our group extended this analysis to 14 astronauts who flew relatively short ∼5‐ to 13‐days Shuttle missions and confirmed the increase of cf‐mtDNA abundance in the plasma of astronauts on the day of return and 3 days after return. Our study also suggests that the release of cf‐mtDNA from mitochondria is associated with the activation of multiple pathways related to inflammation, oxidative stress, and DNA damage. Emerging evidence shows that extracellular vesicles, named exosomes, play a critical role in intercellular communication and regulation of various biological processes, such as proliferation, invasion, metastasis, immune response, and angiogenesis (Regev-Rudzki et al., 2013; Roy et al., 2017; Ohyashiki et al., 2018). Collectively, an increasing body of evidence highlights the importance of exosomes in regulating gene expression.

## Exosomal Long-non-coding RNA in Human Disease

Exosomes are structures defined as small extracellular vesicles measuring 30–140 nm. They are secreted by eukaryotic cells upon fusion of multivesicular bodies with the plasma membrane (Théry et al., 2002). The exosomal cargo contains all types of biomolecules, including bioactive lipids, metabolites, proteins, DNA, messenger RNA (mRNA), and non-coding RNAs such as microRNA and long non-coding RNA (lncRNA) (Jeppesen et al., 2019). Long non-coding RNA (lncRNA) are non-coding transcripts, usually longer than 200 nucleotides, and represent the largest transcript class in the mouse and human transcriptomes. At the molecular level, lncRNA can modulate chromatin structure and function, transcription of neighboring genes, and affect RNA splicing, stability, and translation (Jathar et al., 2017). LncRNAs are also known to activate or repress the transcription of neighboring (cis) or distal (trans) protein-coding genes. Their essential roles in regulating critical molecular and cellular functions make them great candidates for potential clinical biomarkers (Devaux et al., 2015). LncRNAs are implicated in a variety of disease processes, including neurocognitive, cardiovascular diseases, and cancers. In cardiovascular disease, lncRNAs play a role in cardiac hypertrophy, myocardial infarction, heart failure, and pulmonary hypertension (Lozano-Vidal et al., 2019; Bisserier et al., 2020) and have been described as potential biomarkers or therapeutic agents for heart disease, including myocardial infarction, coronary artery disease, heart failure, and diabetic cardiomyopathy (Yang et al., 2014; Rizki and Boyer, 2015). Regarding cancer, several lncRNAs are differentially expressed in breast, colorectal, and colon cancer, as well as melanoma (Kino et al., 2010; Poliseno et al., 2010; Marín-Béjar et al., 2013; Huarte, 2015). Importantly, previous studies showed that exosomal lncRNAs might represent valuable diagnostic markers for any type of cancer. A panel of 21 differentially expressed lncRNAs has been found in exosomes of colorectal cancer (CRC) patients (Dong et al., 2016). Similarly, Zhang et al. found significant upregulation of the lncRNA, Metastasis-associated lung adenocarcinoma transcript 1 (MALAT-1), in exosome isolated from the serum of non-small cell lung cancer (NSCLC) patients (Zhang et al., 2017). Therefore, profiling the exosomal lncRNAs can lead to the identification of promising biomarkers.

Considering their noted implications in various diseases, it is worthwhile to investigate lncRNAs and their role as potential subclinical biomarkers of spaceflight-associated stress. In this context, we analyzed the expression of exosomal lncRNA content by RNA-sequencing (RNA-seq) of exosomes isolated from the blood plasma of three astronauts who flew Shuttle missions between 1998–2001.

## New Insights Into the Role of lncRNA After Spaceflight

Detailed methodology related to astronauts’ samples (spaceflight duration, age, mission ID, storage condition), exosome isolation and characterization, RNA extraction, RNA-sequencing, and bioinformatics pipelines for computational analysis and data visualization is provided in the Supplementary Material.

**FIGURE 1:**
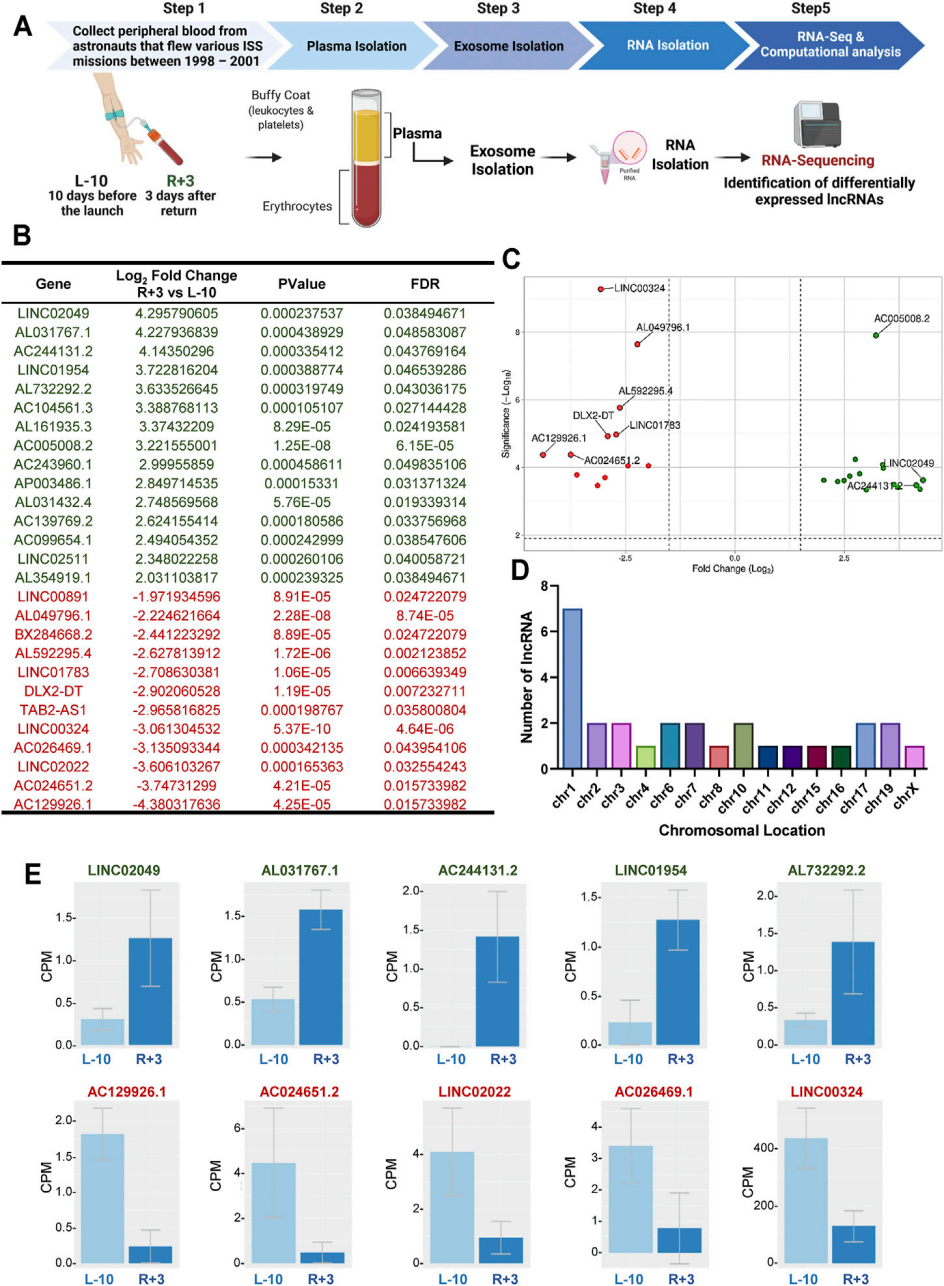
Identification of 27 differentially regulated lncRNA in exosomes from astronauts 3-days post-landing. **(A)** Schematic representation of the experimental design. Blood was sampled at two different time points: 10 days before launch (L-10) and 3 days after return (R+3) from three different astronauts. Exosomes were isolated from blood plasma, and purified exosomal RNA was analyzed by RNA-Sequencing. **(B)** Computational analysis identified 27 differentially regulated lncRNA with a Log_2_ fold change >2, *p* < 0.001, and FDR <0.05, compared to baseline L-10. **(C)** Volcano plots showing Log_2_-fold changes for the 27 differentially regulated lncRNA and the statistical significance of each gene calculated after DEG analysis. Red points indicate significantly down-regulated genes; green points indicate up-regulated genes. **(D)** Chromosomal location of the lncRNA is shown. **(E)** Normalized counts, or the number of reads that align to a particular feature after correcting for sequencing depth and transcriptome composition bias, are shown for the top 5 up-regulated and down-regulated lncRNA in astronauts at L-10 and R+3.

Blood was sampled at two different time points: 10 days before launch (L-10) and 3 days after return (R+3) from three different astronauts who flew relatively short ISS missions (∼5–13-days) between 1998–2001 (Figure 1). Exosomes were isolated from blood plasma at each time point using the ExoQuick^®^ method, as previously described (Bisserier et al., 2021). Exosomes were characterized by nanoparticle tracking analysis for size distribution and concentration, and analyzed using an exosome-specific antibody array. Nanosight and Exo-Check Antibody Array data for the exosomes used in this study were recently published by Bisserier et al. (Bisserier et al., 2021).

We assessed the transcriptome changes associated with spaceflight in the same astronauts at each time point using purified exosomal RNA by RNA-seq, as previously described (Bisserier et al., 2021). Analysis of the RNA-seq datasets identified 27 differentially expressed lncRNAs with a Log_2_ fold change (FC) (*p* < 0.001, and FDR<0.05), with possible molecular and cellular functions, as well as clinical implications (Figure 1B). Of the 27 differentially expressed genes (DEGs), 15 were up-regulated while 12 were down-regulated in R+3 (Figure 1C). Seven of the lncRNA were found on chromosome 1, with the rest being spread evenly across 14 other chromosomes with about 1-2 lncRNA per chromosome (Figure 1D). Normalized counts (i.e., counts per million mapped reads) for the top 5 up-regulated lncRNA genes were LINC02049 (Log_2_FC = 4.29), AL031767.1 (Log_2_FC = 4.22), AC244131.2 (Log_2_FC = 4.14), LINC01954 (Log_2_FC = 3.72), and AL732292.2 (Log_2_FC = 3.63), and the top 5 down-regulated lncRNA genes were AC129926.1 (Log_2_FC = −4.38)**,** AC024651.2 (Log_2_FC = −3.74), LINC02022 (Log_2_FC = 0.00016), AC026469.1 (Log_2_FC = −3.13), and LINC00324 (Log_2_FC = −3.06) (Figure 1E).

**FIGURE 2:**
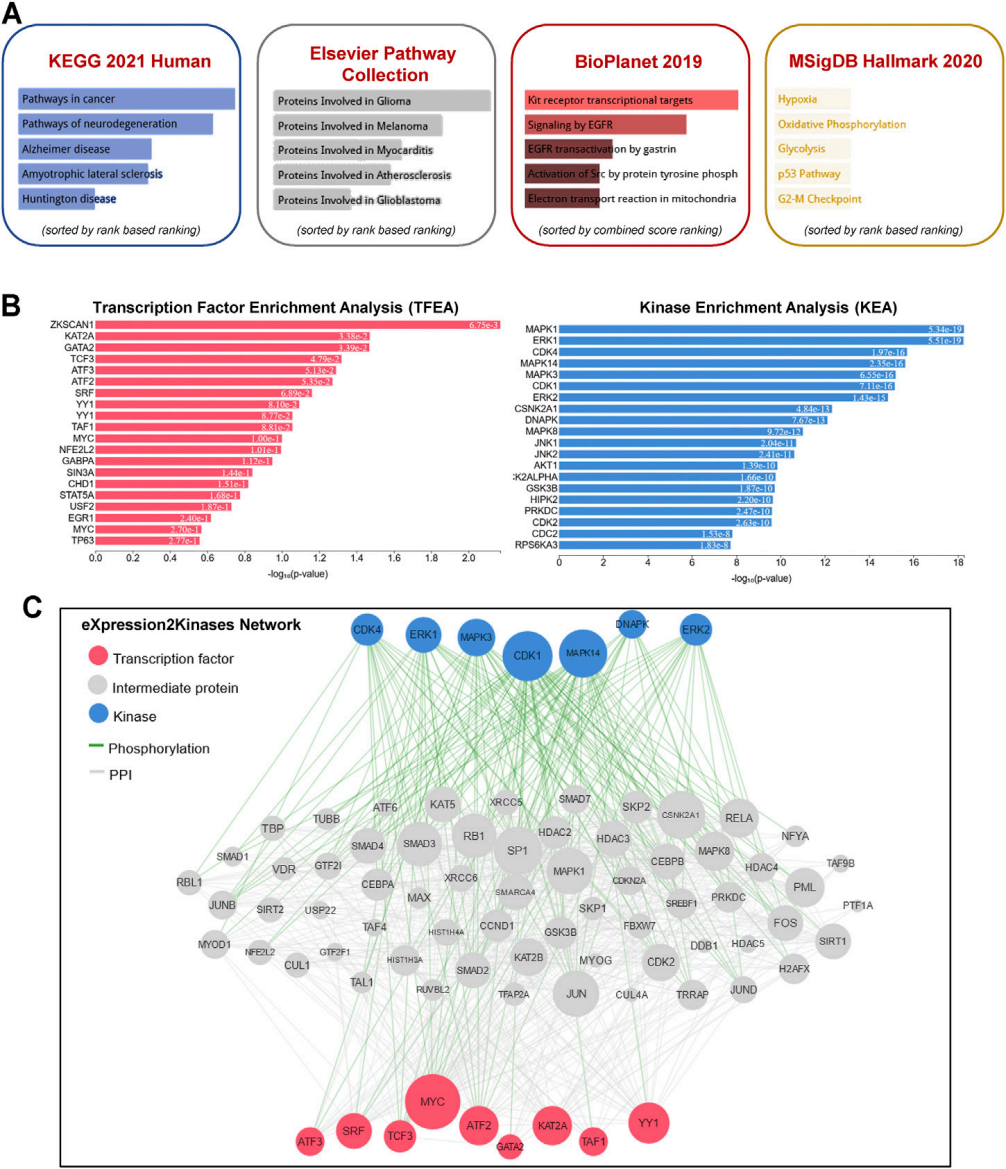
Pathway enrichment analysis and eXpression2Kinases network. **(A)** A comprehensive gene set enrichment analysis for the *cis*- and *trans*-regulatory target genes of differentially expressed lncRNAs was performed using EnrichR 2021 and the 2021 Kyoto Encyclopedia of Genes and Genomes (KEGG) Human database, Elsevier Pathway Collection, BioPlanet 2019, and the Molecular Signatures Database 2020 (MsigDB). **(B)** Transcription factor enrichment analysis (left panel) and kinase enrichment (right panel) analyses are shown for all the *cis-* and *trans-*regulatory target genes of differentially expressed lncRNAs. **(C)** The eXpression2Kinases (X2K) network displays the inferred upstream regulatory network predicted to regulate the input list of genes by integrating the results from the TFEA, the network expansion, and the kinase enrichment. Pink nodes represent the top transcription factors predicted to regulate the expression of the input gene list; blue nodes represent the top predicted protein kinases known to phosphorylate the proteins within the expanded subnetwork. Green network edges/links represent kinase-substrate phosphorylation interactions between kinases and their substrates, while grey network edges represent physical protein-protein interactions.

To gain further insight into the function of the identified lncRNAs, we identified the *cis*- and *trans*-regulatory target genes of the differentially expressed lncRNAs and performed pathway enrichment analysis using the ENRICHR resource (http://amp.pharm.mssm.edu/Enrichr/, accessed 09/2020), an integrative web-based gene list enrichment analysis tool that includes the 2021 Kyoto Encyclopedia of Genes and Genomes (KEGG) Human database, Elsevier Pathway Collection, BioPlanel 2019, and the Molecular Signatures Database 2020 (MsigDB) (Chen et al., 2013; Kuleshov et al., 2016; Xie et al., 2021). Our computational analysis suggested that pathways associated with cancer, neurodegeneration (Alzheimer’s and Huntington’s disease, amyotrophic lateral sclerosis), and cardiovascular disease (myocarditis and atherosclerosis) may be dysregulated (Figure 2A). Interestingly, genes associated with hypoxia, oxidative phosphorylation, glycolysis, p53 pathway, and G2-M checkpoint were also enriched in exosomes isolated at R+3. Next, we used the eXpression2Kinases Network (X2K) Web platform to understand upstream regulatory networks from signatures of differentially expressed genes (Clarke et al., 2018). By combining transcription factor enrichment analysis and protein-protein interaction network expansion with kinase enrichment analysis, we generated inferred networks of transcription factors, proteins, and kinases predicted to regulate the expression of the identified lncRNA-targeted genes. Transcription factor enrichment analysis identified the following top 5 transcriptional regulators - ZKSCAN1, KAT2A, GATA2, TCF3, and ATF3 (Figure 2B, left panel). Similarly, kinase enrichment analysis identified MAPK1, ERK1, CDK4, MAPK14, and CDK1 (top 5) (Figure 2B, right panel). Integration of the TFEA and KEA analysis allowed us to build the eXpression2Kinases Network (Figure 2C) and displays the inferred upstream regulatory network predicted to regulate the lncRNA-targeted “*Cis*” and “*Trans*” genes (Clarke et al., 2018). Next, we used a human lncRNA database called LncSEA, to screen for lncRNAs related to prognosis. Each cancer survival-related lncRNA was characterized using clinical data from The Cancer Genome Atlas project and univariate Cox regression (Table 1). Further analysis of the 27-differentially regulated lncRNAs revealed only LINC00324, LINC00891, LINC01783, LINC01954, LINC02022, LINC02049, and LINC02511 might have prognostic survival value in cancer patients. Given lncRNAs often function as competing endogenous RNAs-binding miRNA family members, lncRNA-miRNA interactions play a critical role in regulating gene expression and biological processes. Through integrating miRNA-target interactions in large-scale CLIP-Seq (HITS-CLIP, PAR-CLIP, iCLIP, CLASH) data from StarBase2.0 and LncBase2.0, our result showed that only LINC00324 might potentially interact with a broad range of miRNA (Table 2).

**TABLE 1:** LncRNA related to prognosis using TCGA project. Differentially regulated lncRNAs in astronauts’ exosomes at R+3 were further analyzed using the LncSEA database that integrates clinical data from The Cancer Genome Atlas project to screen for lncRNAs related to prognosis after univariate Cox regression analysis.

NA	Class	Gene	Count
LINC00324	Survival	UCEC	2049
	Survival	BLCA	2135
	Survival	CESC	1433
	Survival	HNSC	1530
	Survival	LGG	5017
	Survival	LIHC	2296
	Survival	LUAD	1703
	Survival	MESO	1749
	Survival	HNSC	1530
	Survival	KICH	2806
LINC00891	Survival	KIRP	3699
	Survival	LUAD	1703
	Survival	SARC	1571
	Survival	LGG	5017
	Survival	LIHC	2296
LINC01783	Survival	PAAD	3191
	Survival	DLBC	561
	Survival	KIRC	3997
	Survival	LIHC	2296
	Survival	LUAD	1703
LINC01954	Survival	PAAD	3191
	Survival	UVM	2175
	Survival	KIRC	3997
	Survival	LAML	2191
LINC02022	Survival	KICH	2806
LINC02049	Survival	BRCA	1581
	Survival	LIHC	2296
	Survival	SARC	1571
LINC02511	Survival	KIRC	3997
	Survival	KIRP	3699
	Survival	LIHC	2296

**TABLE 2:** Predicted LncRNA-miRNA interactions. miRNA-target interactions were analyzed for the 27 lncRNA using large-scale CLIP-Seq (HITS-CLIP, PAR-CLIP, iCLIP, CLASH) data from StarBase2.0 and LncBase2.0. Only LINC00324 was identified and predicted to interact with other miRNAs.

LncRNA	microRNA	Count
LINC00324	hsa-miR-25-5p	10
	hsa-miR-676-3p	62
	hsa-miR-3175	10
	hsa-miR-542-3p	72
	hsa-miR-542-5p	11
	hsa-miR-1226-3p	100
	hsa-miR-663a	8
	hsa-miR-744-5p	65
	hsa-miR-92a-2-5p	12
	hsa-miR-197-3p	114
	hsa-miR-181a-2-3p	79
	hsa-miR-625-3p	26

## Discussion

Ongoing research aims to better understand and define the spaceflight-related health risks in astronauts. In this context, identifying new non-invasive screening tools and methods such as new biomarkers for early detection and prognostication for cancer, neurodegenerative, and cardiovascular diseases is of great clinical value. Over the past years, lncRNAs have emerged as promising diagnostic or prognostic biomarkers for various diseases (Sun et al., 2016; Bolha et al., 2017). Here, we identified 27 differentially expressed lncRNAs by RNA-seq in exosomes isolated from the blood plasma of three astronauts who flew short ISS missions as early as 3 days post-landing. Interestingly, our computational analysis indicates that the lncRNA-target genes may regulate critical biological functions and have clinical implications. Furthermore, 7 out of the 27 initially identified lncRNAs may have a prognostic value in cancer. These lncRNAs are dysregulated as early as 3 days after landing (R+3). Therefore, our result may lay a foundation for identifying new biomarkers associated with space travel, which may allow for early therapeutic interventions that may improve astronauts’ quality of life and life expectancy. In addition, a large body of scientific evidence suggests that lncRNAs regulate various cell processes, including transcription, intracellular trafficking, and chromosome remodeling (Rinn and Chang, 2012; Quinn and Chang, 2016). As a result, lncRNA dysregulation may induce aberrant gene expression and trigger the development of cancer, which emphasizes the urgent need to characterize the functional role of these lncRNAs. Consistent with our results, a few recent reports showed that lncRNA expression is affected by irradiation and microgravity (Hu et al., 2017; Fu et al., 2020). In the study conducted by Fu et al., specific lncRNAs were significantly down- or up-regulated in human lymphoblastoid TK6 cells exposed to static or rotating conditions to simulate microgravity in space or 2-Gy *γ*-ray irradiation. In the group exposed to both microgravity and radiation, 21.4% of lncRNAs were down-regulated and 78.6% were up-regulated (Fu et al., 2020). Further analysis of the transcriptome profile identified increased expression of differentially expressed genes associated with inflammatory (LPS/TLR, TNF, NF-κB) and p53 signaling pathways (Fu et al., 2020). Overall, their data showed that genes involved in inflammatory response were almost all up-regulated under microgravity and radiation conditions. Given the critical role of lncRNA in the transcriptional and post-transcriptional regulation of protein-coding genes, they represent an emerging area of focus in molecular biology.

There are a few limitations worth mentioning. First, we used a stringent cutoff threshold (Log_2_FC > 2, *p* < 0.001), which could exclude other lncRNAs with a *p*-value between 0.001 and 0.05 that may also have an important role in cancer, as well as neurocognitive and cardiovascular diseases. Second, our analysis was performed using exosomal RNA isolated only from three astronauts due to the exceptionally rare availability of such samples. Finally, the very restricted access to the clinical data from these astronauts does not allow us to extrapolate possible short-term post-flight correlations (weeks and months) and, more importantly, longitudinal (1–20 years) clinical follow-up studies, as the blood samples were collected more than 20 years ago. Therefore, further investigations using a controlled environment that experimentally replicates the multiple stressors of the space environment, such as space radiation or microgravity, are required to better characterize the role and prognostic value of lncRNAs on adverse health risks. Overall, we aimed to provide new insights into the diagnostic and prognostic value of astronauts-derived exosomal lncRNAs as emerging biomarkers and highlight the potential role of exosomal lncRNA in health risks associated with spaceflight.

## Data Availability

The raw data supporting the conclusions of this article will be made available by the authors, without undue reservation.

## References

[B1] Amidon G. L. DeBrincat G. A. Najib N. (1991). Effects of Gravity on Gastric Emptying, Intestinal Transit, and Drug Absorption. J. Clin. Pharmacol. 31, 968–973. 10.1002/j.1552-4604.1991.tb03658.x 1761729

[B2] Barrila J. Ott C. M. LeBlanc C. Mehta S. K. Crabbé A. Stafford P. (2016). Spaceflight Modulates Gene Expression in the Whole Blood of Astronauts. NPJ Microgravity 2, 16039. 10.1038/npjmgrav.2016.39 28725744 PMC5515525

[B3] Bisserier M. Shanmughapriya S. Rai A. K. Gonzalez C. Brojakowska A. Garikipati V. N. S. (2021). Cell-Free Mitochondrial DNA as a Potential Biomarker for Astronauts' Health. J. Am. Heart Assoc. 10, e022055. 10.1161/JAHA.121.022055 34666498 PMC8751818

[B4] Bisserier M. Janostiak R. Lezoualc’h F. Hadri L. (2020). Targeting Epigenetic Mechanisms as an Emerging Therapeutic Strategy in Pulmonary Hypertension Disease. Vasc. Biol. 2, R17–R34. 10.1530/vb-19-0030 32161845 PMC7065685

[B5] Bolha L. Ravnik-Glavač M. Glavač D. (2017). Long Noncoding RNAs as Biomarkers in Cancer. Dis. Markers 2017, 7243968. 10.1155/2017/7243968 28634418 PMC5467329

[B6] Budinger T. F. Lyman J. T. Tobias C. A. (1972). Visual Perception of Accelerated Nitrogen Nuclei Interacting with the Human Retina. Nature 239, 209–211. 10.1038/239209a0 4562730

[B7] Carmeliet G. Bouillon R. (1999). The Effect of Microgravity on Morphology and Gene Expression of Osteoblasts *In Vitro* . FASEB J. 13 (Supp. l), S129–S134. 10.1096/fasebj.13.9001.s129 10352154

[B8] Cekanaviciute E. Rosi S. Costes S. V. (2018). Central Nervous System Responses to Simulated Galactic Cosmic Rays. Int. J. Mol. Sci. 19. 10.3390/ijms19113669 PMC6275046 30463349

[B9] Chancellor J. Scott G. Sutton J. (2014). Space Radiation: The Number One Risk to Astronaut Health beyond Low Earth Orbit. Life 4, 491–510. 10.3390/life4030491 25370382 PMC4206856

[B10] Chen E. Y. Tan C. M. Kou Y. Duan Q. Wang Z. Meirelles G. V. (2013). Enrichr: Interactive and Collaborative HTML5 Gene List Enrichment Analysis Tool. BMC Bioinformatics 14, 128. 10.1186/1471-2105-14-128 23586463 PMC3637064

[B11] Clarke D. J. B. Kuleshov M. V. Schilder B. M. Torre D. Duffy M. E. Keenan A. B. (2018). eXpression2Kinases (X2K) Web: Linking Expression Signatures to Upstream Cell Signaling Networks. Nucleic Acids Res. 46, W171–W179. 10.1093/nar/gky458 29800326 PMC6030863

[B12] Coleman M. A. Sasi S. P. Onufrak J. Natarajan M. Manickam K. Schwab J. (2015). Low-dose Radiation Affects Cardiac Physiology: Gene Networks and Molecular Signaling in Cardiomyocytes. Am. J. Physiology-Heart Circulatory Physiol. 309, H1947–H1963. 10.1152/ajpheart.00050.2015 PMC4698384 26408534

[B13] Cucinotta F. A. Manuel F. K. Jones J. Iszard G. Murrey J. Djojonegro B. (2001). Space Radiation and Cataracts in Astronauts. Radiat. Res. 156, 460–466. 10.1667/0033-7587(2001)156[0460:sracia]2.0.co;2 11604058

[B14] Delp M. D. Charvat J. M. Limoli C. L. Globus R. K. Ghosh P. (2016). Apollo Lunar Astronauts Show Higher Cardiovascular Disease Mortality: Possible Deep Space Radiation Effects on the Vascular Endothelium. Sci. Rep. 6, 29901. 10.1038/srep29901 27467019 PMC4964660

[B15] Devaux Y. Zangrando J. Schroen B. Creemers E. E. Pedrazzini T. Chang C. P. (2015). Long Noncoding RNAs in Cardiac Development and Ageing. Nat. Rev. Cardiol. 12, 415–425. 10.1038/nrcardio.2015.55 25855606

[B16] Dong L. Lin W. Qi P. Xu M.-d. Wu X. Ni S. (2016). Circulating Long RNAs in Serum Extracellular Vesicles: Their Characterization and Potential Application as Biomarkers for Diagnosis of Colorectal Cancer. Cancer Epidemiol. Biomarkers Prev. 25, 1158–1166. 10.1158/1055-9965.epi-16-0006 27197301

[B17] Du J. Cui J. Yang J. Wang P. Zhang L. Luo B. (2021). Alterations in Cerebral Hemodynamics during Microgravity: A Literature Review. Med. Sci. Monit. 27, e928108. 10.12659/MSM.928108 33446627 PMC7814510

[B18] Fu H. Su F. Zhu J. Zheng X. Ge C. (2020). Effect of Simulated Microgravity and Ionizing Radiation on Expression Profiles of miRNA, lncRNA, and mRNA in Human Lymphoblastoid Cells. Life Sci. Space Res. 24, 1–8. 10.1016/j.lssr.2019.10.009 31987473

[B19] Garikipati V. N. S. Arakelyan A. Blakely E. A. Chang P. Y. Truongcao M. M. Cimini M. (2021). Long-Term Effects of Very Low Dose Particle Radiation on Gene Expression in the Heart: Degenerative Disease Risks. Cells 10. 10.3390/cells10020387 PMC7917872 33668521

[B20] Garrett-Bakelman F. E. Darshi M. Green S. J. Gur R. C. Lin L. Macias B. R. (2019). The NASA Twins Study: A Multidimensional Analysis of a Year-Long Human Spaceflight. Science 364. 10.1126/science.aau8650 PMC7580864 30975860

[B21] Grimm D. Grosse J. Wehland M. Mann V. Reseland J. E. Sundaresan A. (2016). The Impact of Microgravity on Bone in Humans. Bone 87, 44–56. 10.1016/j.bone.2015.12.057 27032715

[B22] Hu Z. Wang H. Wang Y. Zhou H. Shi F. Zhao J. (2017). Genome-wide Analysis and Prediction of Functional Long Noncoding RNAs in Osteoblast Differentiation under Simulated Microgravity. Mol. Med. Rep. 16, 8180–8188. 10.3892/mmr.2017.7671 28990099 PMC5779904

[B23] Huarte M. (2015). The Emerging Role of lncRNAs in Cancer. Nat. Med. 21, 1253–1261. 10.1038/nm.3981 26540387

[B24] Jathar S. Kumar V. Srivastava J. Tripathi V. (2017). Technological Developments in lncRNA Biology. Adv. Exp. Med. Biol. 1008, 283–323. 10.1007/978-981-10-5203-3_10 28815544

[B25] Jeppesen D. K. Fenix A. M. Franklin J. L. Higginbotham J. N. Zhang Q. Zimmerman L. J. (2019). Reassessment of Exosome Composition. Cell 177, 428–445. 10.1016/j.cell.2019.02.029 30951670 PMC6664447

[B26] Kino T. Hurt D. E. Ichijo T. Nader N. Chrousos G. P. (2010). Noncoding RNA Gas5 Is a Growth Arrest- and Starvation-Associated Repressor of the Glucocorticoid Receptor. Sci. Signal. 3, ra8. 10.1126/scisignal.2000568 20124551 PMC2819218

[B27] Kuleshov M. V. Jones M. R. Rouillard A. D. Fernandez N. F. Duan Q. Wang Z. (2016). Enrichr: a Comprehensive Gene Set Enrichment Analysis Web Server 2016 Update. Nucleic Acids Res. 44, W90–W97. 10.1093/nar/gkw377 27141961 PMC4987924

[B28] Lawley J. S. Petersen L. G. Howden E. J. Sarma S. Cornwell W. K. Zhang R. (2017). Effect of Gravity and Microgravity on Intracranial Pressure. J. Physiol. 595, 2115–2127. 10.1113/jp273557 28092926 PMC5350445

[B29] Lozano-Vidal N. Bink D. I. Boon R. A. (2019). Long Noncoding RNA in Cardiac Aging and Disease. J. Mol. Cel Biol 11, 860–867. 10.1093/jmcb/mjz046 PMC6884711 31152659

[B30] Marín-Béjar O. Marchese F. P. Athie A. Sánchez Y. González J. Segura V. (2013). Pint lincRNA Connects the P53 Pathway with Epigenetic Silencing by the Polycomb Repressive Complex 2. Genome Biol. 14, R104. 10.1186/gb-2013-14-9-r104 24070194 PMC4053822

[B31] Mehta S. K. Bloom D. C. Plante I. Stowe R. Feiveson A. H. Renner A. (2018). Reactivation of Latent Epstein-Barr Virus: A Comparison after Exposure to Gamma, Proton, Carbon, and Iron Radiation. Int. J. Mol. Sci. 19. 10.3390/ijms19102961 PMC6213004 30274169

[B32] Mewaldt R. A. (1994). Galactic Cosmic ray Composition and Energy Spectra. Adv. Space Res. 14, 737–747. 10.1016/0273-1177(94)90536-3 11540019

[B33] Moreno-Villanueva M. Wong M. Lu T. Zhang Y. Wu H. (2017). Interplay of Space Radiation and Microgravity in DNA Damage and DNA Damage Response. NPJ Microgravity 3, 14. 10.1038/s41526-017-0019-7 28649636 PMC5460239

[B34] Ohyashiki J. H. Umezu T. Ohyashiki K. (2018). Extracellular Vesicle-Mediated Cell-Cell Communication in Haematological Neoplasms. Philos. Trans. R. Soc. Lond. B Biol. Sci. 373. 10.1098/rstb.2016.0484 PMC5717438 29158313

[B35] Okada A. Ichikawa J. Tozawa K. (2011). Kidney Stone Formation during Space Flight and Long-Term Bed Rest. Clin. Calcium 21, 1505–1510. 21960236

[B36] Payne M. W. C. Williams D. R. Trudel G. (2007). Space Flight Rehabilitation. Am. J. Phys. Med. Rehabil. 86, 583–591. 10.1097/phm.0b013e31802b8d09 17167347

[B37] Poliseno L. Salmena L. Zhang J. Carver B. Haveman W. J. Pandolfi P. P. (2010). A Coding-independent Function of Gene and Pseudogene mRNAs Regulates Tumour Biology. Nature 465, 1033–1038. 10.1038/nature09144 20577206 PMC3206313

[B38] Quinn J. J. Chang H. Y. (2016). Unique Features of Long Non-coding RNA Biogenesis and Function. Nat. Rev. Genet. 17, 47–62. 10.1038/nrg.2015.10 26666209

[B39] Regev-Rudzki N. Wilson D. W. Carvalho T. G. Sisquella X. Coleman B. M. Rug M. (2013). Cell-cell Communication between Malaria-Infected Red Blood Cells *via* Exosome-like Vesicles. Cell 153, 1120–1133. 10.1016/j.cell.2013.04.029 23683579

[B40] Rinn J. L. Chang H. Y. (2012). Genome Regulation by Long Noncoding RNAs. Annu. Rev. Biochem. 81, 145–166. 10.1146/annurev-biochem-051410-092902 22663078 PMC3858397

[B41] Rizki G. Boyer L. A. (2015). Lnc Ing Epigenetic Control of Transcription to Cardiovascular Development and Disease. Circ. Res. 117, 192–206. 10.1161/circresaha.117.304156 26139858

[B42] Roy S. Kim D. Lim R. (2017). Cell-cell Communication in Diabetic Retinopathy. Vis. Res. 139, 115–122. 10.1016/j.visres.2017.04.014 28583293 PMC5723213

[B43] Shen M. Frishman W. H. (2019). Effects of Spaceflight on Cardiovascular Physiology and Health. Cardiol. Rev. 27, 122–126. 10.1097/crd.0000000000000236 30365406

[B44] Siddiqui R. Akbar N. Khan N. A. (2021). Gut Microbiome and Human Health under the Space Environment. J. Appl. Microbiol. 130, 14–24. 10.1111/jam.14789 32692438

[B45] Sonnenfeld G. (2002). The Immune System in Space and Microgravity. Med. Sci. Sports Exerc. 34, 2021–2027. 10.1097/00005768-200212000-00024 12471311

[B46] Sun C. Jiang H. Sun Z. Gui Y. Xia H. (2016). Identification of Long Non-coding RNAs Biomarkers for Early Diagnosis of Myocardial Infarction from the Dysregulated Coding-Non-Coding Co-expression Network. Oncotarget 7, 73541–73551. 10.18632/oncotarget.11999 27634901 PMC5341997

[B47] Tanaka K. Nishimura N. Kawai Y. (2017). Adaptation to Microgravity, Deconditioning, and Countermeasures. J. Physiol. Sci. 67, 271–281. 10.1007/s12576-016-0514-8 28000175 PMC10717636

[B48] Taylor W. E. Bhasin S. Lalani R. Datta A. Gonzalez-Cadavid N. F. (2002). Alteration of Gene Expression Profiles in Skeletal Muscle of Rats Exposed to Microgravity during a Spaceflight. J. Gravit. Physiol. 9, 61–70. 14638460

[B49] Théry C. Zitvogel L. Amigorena S. (2002). Exosomes: Composition, Biogenesis and Function. Nat. Rev. Immunol. 2, 569–579. 10.1038/nri855 12154376

[B50] Vernice N. A. Meydan C. Afshinnekoo E. Mason C. E. (2020). Long-term Spaceflight and the Cardiovascular System. Precis Clin. Med. 3, 284–291. 10.1093/pcmedi/pbaa022 33391848 PMC7757439

[B51] Whitney A. R. Diehn M. Popper S. J. Alizadeh A. A. Boldrick J. C. Relman D. A. (2003). Individuality and Variation in Gene Expression Patterns in Human Blood. Proc. Natl. Acad. Sci. 100, 1896–1901. 10.1073/pnas.252784499 12578971 PMC149930

[B52] Xie Z. Bailey A. Kuleshov M. V. Clarke D. J. B. Evangelista J. E. Jenkins S. L. (2021). Gene Set Knowledge Discovery with Enrichr. Curr. Protoc. 1, e90. 10.1002/cpz1.90 33780170 PMC8152575

[B53] Yang K.-C. Yamada K. A. Patel A. Y. Topkara V. K. George I. Cheema F. H. (2014). Deep RNA Sequencing Reveals Dynamic Regulation of Myocardial Noncoding RNAs in Failing Human Heart and Remodeling with Mechanical Circulatory Support. Circulation 129, 1009–1021. 10.1161/circulationaha.113.003863 24429688 PMC3967509

[B54] Yatagai F. Honma M. Dohmae N. Ishioka N. (2019). Biological Effects of Space Environmental Factors: A Possible Interaction between Space Radiation and Microgravity. Life Sci. Space Res. 20, 113–123. 10.1016/j.lssr.2018.10.004 30797428

[B55] Zhang R. Xia Y. Wang Z. Zheng J. Chen Y. Li X. (2017). Serum Long Non Coding RNA MALAT-1 Protected by Exosomes Is Up-Regulated and Promotes Cell Proliferation and Migration in Non-small Cell Lung Cancer. Biochem. Biophysical Res. Commun. 490, 406–414. 10.1016/j.bbrc.2017.06.055 28623135

[B56] Zobel B. B. Del Vescovo R. Oliva G. Russo V. Setola R. (2012). Assessing Bone Loss in Micro-gravity: A Fuzzy Approach. Comp. Methods Programs Biomed. 108, 910–921. 10.1016/j.cmpb.2012.05.001 22727257

