# Supplementary material for: Emerging Role of Exosomal Long Non-coding RNAs in Spaceflight-Associated Risks in Astronauts
Source: Front Genet. 2022 Jan 17;12:812188. doi: 10.3389/fgene.2021.812188 (PMC8803151; doi:10.3389/fgene.2021.812188)
Supplement: Supplementary file 1 [file Table1.pdf]

SUPPLEMENTAL MATERIAL

The data that support the findings of this study are available from the corresponding author upon reasonable request. This study was approved by NASA and Icahn School of Medicine at Mount Sinai's Institutional Review Board (STUDY00000075 and HSM19-00367, respectively). All study participants provided written informed consent at the time of sample collection.

Astronaut samples

Blood was sampled 10 days before launch (L-10) and 3 days after return (R+3) from 3 astronauts who flew short (up to 14 days) Shuttle missions between 1998 and 2001. Information regarding de-identified blood samples, including Shuttle Space mission code, the approximate average age of the crew members, and time spent in space, are provided in the table below. All samples were stored at -80°C until use.

Sample ID #	Binned Age at Launch in Years
C1	≥ 50
C2	< 40
C3	< 40

Thrombin plasma preparation for exosome precipitation

Exosomes were isolated from blood plasma samples of 3 astronauts (C1, C2, and C3) at L-10, and R+3 using the ExoQuick Plasma prep and Exosome precipitation kit (Cat # EXOQ5TM, System Biosciences, CA, USA), as previously described (1). Briefly, thrombin was added to the plasma and incubated for 5 min at room temperature. Next, samples were centrifuged at 10,000 rpm for 5 min, and the supernatant was incubated with the exosome precipitation solution for 30 minutes at 4° C. Samples were centrifuged for 30 minutes at 1,500 x g at 4° C. Finally, the supernatant was aspirated, and the beige-colored pellet was dissolved in 100 μl of sterile 1x PBS.

Exosome antibody array

EVs were characterized by nanoparticle tracking analysis (Nanosight). EV population was further validated using Exo-Check™ Exosome Antibody Array (Cat # EXORAY210A-8, System Biosciences, CA, USA). Briefly, 50 μg of protein were incubated with the labeling reagent for 30

min at RT, and excess labeling reagent was removed according to the manufacturer's protocol. After blocking, the membrane was incubated overnight with the labeled exosomes at 4º C. Then, the membrane was washed for 5 min at RT and incubated with the detection buffer for 30 minutes. The membrane was washed 3 times for 5 min using the wash buffer and developed using the chemiluminescence detection system (Clarity Western ECL substrate, cat # 170-5060S, Bio-Rad, USA).

RNA isolation

Total RNA was extracted from astronaut-derived exosomes using blood collected at L-10 and R+3 using the RNeasy Mini Kit (Qiagen), according to the manufacturer's protocol. High-quality RNA was isolated using RNeasy Mini spin columns and eluted using molecular-grade water (RNAse/DNAse free). All samples first undergo quality control assessment to ensure successful library preparation and sequencing. RNA sample quality was assessed by NanoDrop and Agilent 2100 BioAnalyzer. Samples with RNA integrity number (RIN) above 7, OD260/280: 2, and OD260/230 ≥ 2 were used. In addition, RNA degradation and contamination were monitored on 1% agarose gels before RNA-Seq.

Library preparation and small RNA sequencing

RNA quality was assessed using an Agilent TapeStation (Agilent, Palo Alto, CA, USA), and RNA concentration was quantified by Qubit 4.0 spectrophotometer. The library for small RNA sequencing was prepared using the Smarter smRNA-seq kit for Illumina (Takara Bio Inc., USA). The quantity and quality of amplified libraries were evaluated using Qubit (Invitrogen, Carlsbad, CA, USA) and Agilent TapeStation high sensitivity D1000 Screen Tape. Small RNA-seq libraries were sequenced using single-end 75 base pairs (PE75) sequencing chemistry on NextSeq 500 instruments following the manufacturer's protocols (Illumina).

Sequencing data analysis

Raw Fastq files were trimmed using cutadapt and built-in Illumina adapters. The quality of trimmed reads was assessed with FastQC, which is freely available at https://www.bioinformatics.babraham.ac.uk/projects/fastqc. Reads were aligned to human genome reference build GRCh38/hg38 with STAR aligner allowing for reads multi-mapping to

different parts of the genome. GENCODE release 38 was used for gene and transcript annotations. Raw counts were normalized by library size and transformed to log2 with edgeR package. Data analysis pipeline is depicted graphically below.

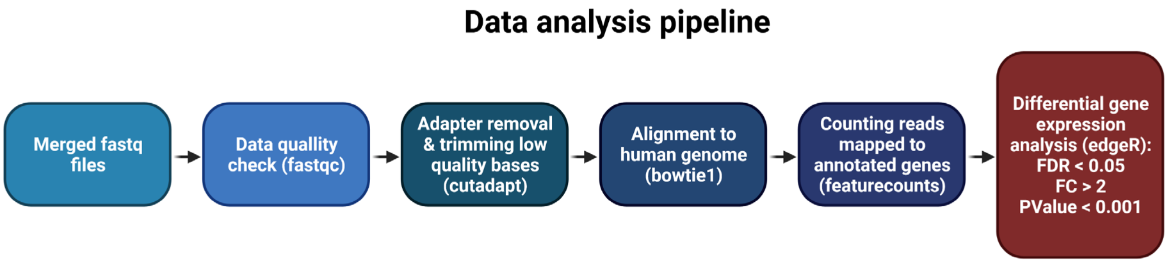

Data visualization and Gene Enrichment Analyses

Volcano plots are used to infer the overall distribution of differentially expressed lncRNA. Log2-fold changes and statistical significance of each gene were calculated. Gene enrichment analysis was performed using Cis and Trans-genes of differentially expressed lncRNAs using the ENRICHR resource (http://amp.pharm.mssm.edu/Enrichr/, accessed 09/2020), an integrative web-based gene list enrichment analysis tool that includes the 2021 Kyoto Encyclopedia of Genes and Genomes (KEGG) Human database, Elsevier Pathway Collection, BioPlanet 2019, and MSigDB Hallmark 2020 database.

REFERENCES

1. Bisserier M, Shanmughapriya S, Rai AK, Gonzalez C, Brojakowska A, Garikipati VNS, et al. Cell-Free Mitochondrial DNA as a Potential Biomarker for Astronauts' Health. *J Am Heart Assoc* (2021):e022055. Epub 2021/10/21. doi: 10.1161/JAHA.121.022055. PubMed PMID: 34666498.
